# The measurement of physical functioning among patients with Tenosynovial Giant Cell Tumor (TGCT) using the Patient-Reported Outcomes Measurement Information System (PROMIS)

**DOI:** 10.1186/s41687-019-0099-0

**Published:** 2019-02-04

**Authors:** Heather L. Gelhorn, Xin Ye, Rebecca M. Speck, Sandra Tong, John H. Healey, Susan V. Bukata, Richard D. Lackman, Lindsey Murray, Grant Maclaine, William R. Lenderking, Henry H. Hsu, Paul S. Lin, William D. Tap

**Affiliations:** 1 Evidera, 7101 Wisconsin Avenue, Suite 1400, Bethesda, MD 20814 USA; 2 Daiichi Sankyo Pharma Development, 399 Thornall St, Edison, NJ 08837 USA; 3 Plexxikon Inc., 91 Bolivar Dr, Berkeley, CA 94710 USA; 4 Memorial Sloan Kettering Cancer Center and Weill Cornell Medical College, 1300 York Ave, New York, NY 10065 USA; 5 UCLA, Los Angeles, CA 90095 USA; 6 Cooper University Health Center, 1 Cooper Plaza, Camden, NJ 08103 USA; 7 Daiichi Sankyo UK Ltd., Chiltern Place, 1 Chalfont Park, Chalfont St Peter, Gerrards Cross, SL9 0GA UK

**Keywords:** Qualitative, Item response theory, Physical functioning, Rare disease, TGCT, PROMIS

## Abstract

**Background:**

Tenosynovial giant cell tumor (TGCT), a rare, locally aggressive neoplasm of the synovium of joints and tendon sheaths, is associated with joint destruction, pain and swelling. Impacts on physical function (PF) vary depending on tumor size and location. The aim of this study was to identify relevant items, and demonstrate the content validity of custom measures of lower extremity PF from the Patient-Reported Outcomes Measurement Information System Physical Function Physical Function (PROMIS-PF) item bank among patients with TGCT.

**Methods:**

Patients were recruited for qualitative research interviews to identify predominant TGCT symptoms and impacts. Patients completed a checklist to evaluate the relevance of each PROMIS-PF item. The publicly available PROMIS-PF item response theory (IRT) parameters were used to select items representing the range of the latent PF trait.

**Results:**

Participants (*n* = 20) were 75% female, mean age 42.5 years. TGCTs were located in the knee (*n* = 15), hip (*n* = 3), and ankle (*n* = 2). Fifty-four PROMIS-PF items were identified as relevant by ≥20% of the participants. PF concepts discussed by participants during the qualitative interviews were also used to select relevant items. Selected items (*n* = 13) were used to create a physical function subscale specific to lower extremity tumors.

**Conclusions:**

We describe a novel method of combining qualitative research and IRT-based item information to select a relevant and content valid subset of PROMIS-PF items to assess heterogeneous impacts on PF in TGCT, a rare disease population.

**Electronic supplementary material:**

The online version of this article (10.1186/s41687-019-0099-0) contains supplementary material, which is available to authorized users.

## Background

Pigmented villonodular synovitis (PVNS) and giant cell tumors of the tendon sheath (GCT-TS) are members of a single condition referred to as “tenosynovial giant cell tumor (TGCT), diffuse and localized type” and have a common pathogenesis. TGCT are rare neoplasms that may result in life-altering functional limitations, morbidity, and diminished patient quality of life (QOL), particularly in recurrent or refractory disease [1, 2]. The current standard of care for TGCT is surgical resection of the tumor as completely as possible in order to (i) reduce symptoms and joint destruction, (ii) improve function, and (iii) minimize the risk of recurrence [3]. However, medical therapies may be on the horizon. It has been observed that most TGCT tumors are associated with elevated expression of the colony-stimulating factor 1 (CSF1) gene [4] and may be driven by a CSF1 gene translocation [5, 6]. This has led to the development of non-surgical, targeted therapies against the CSF1 receptor (CSF1R) where regression in tumor volume is the primary indicator of response, and chronic therapy and monitoring may be indicated [2, 7].

Patient-reported outcome (PRO) assessments of symptoms and health-related quality of life (HRQL) are important in order to support the relevance of primary endpoints which are clinical in nature [8], such as the tumor volume response studied in TGCT. In addition, the Food and Drug Administration (FDA) has explicitly asked for endpoints, like PRO assessments, to support the relevance of progression-free survival [9]. This may be particularly pertinent to TGCT, where chronic therapy with systemic drugs may be accompanied by prolonged risk for potential drug-related toxicities. In contrast, duration of exposure to systemic agents is limited by a patient’s lifespan when treating tumors with high mortality [10].

Due to the dearth of research in the area, a qualitative interview study was conducted to identify and characterize the symptoms of TGCT from the patient perspective [11]. In addition, the content validity of several PRO instruments that might appropriately assess these symptoms in the context of a subsequent clinical trial was evaluated. Hypothesizing that physical functioning would be important and relevant, a primary instrument selected for content validation in the qualitative interview study included the Patient-Reported Outcomes Measurement Information System Physical Function (PROMIS-PF) items. The PROMIS-PF scale emerged as the most appropriate for evaluation of physical functioning in the TGCT population primarily because it includes a wide range of relevant items that target both upper and lower extremity limitations [11].

### PROMIS instrument

The PROMIS-PF is a self-administered 121 item bank that includes questions to assess physical functioning. This PROMIS PF item bank served as the source of items that were selected for the measurement of physical functioning in this study. This approach was taken because PROMIS offers a breadth of item options, and these items were developed and validated using very rigorous methods.

The process for the development of all PROMIS items, including the PROMIS-PF item bank, has been well documented [12–15]. Six phases of Qualitative Item Review for item development were undertaken and included: identification of extant items, item classification and selection, item review and revision, focus group input on domain coverage, cognitive interviews on individual items, and final revision before field testing [13]. Item response theory (IRT)-based analysis of 11 large datasets supplemented and informed item-level qualitative review of nearly 7000 items from available PRO measures in the item library [12]. The details pertaining to the development of the PROMIS-PF item bank, have been described elsewhere [14, 15]. There are four domains in the PROMIS PF item bank: mobility (lower extremity), dexterity (upper extremity), axial (neck and back function) and complex activities that involve more than one subdomain. All items include a Likert response scale with higher scores representing better physical functioning.

There have been no prior studies on the use of PROMIS-PF items among patients with TGCT. However, there are some peer-reviewed publications describing content validity work in lower extremity orthopedic [16, 17] and arthritis patient populations [18]. This prior work is relevant because patients with these diseases experience similar symptoms and impacts to those experienced by patients with TGCT. Hung and colleagues [16] enrolled 382 outpatient orthopedic patients with lower extremity disorders with a goal of developing a lower extremity physical functioning computer adaptive test (CAT) based on the PROMIS-PF item bank. Methods included a qualitative review and psychometric analyses, including real data CAT simulations [16]. The resulting 79-item lower extremity physical function item bank was found to be unidimensional and free of item bias, demonstrating high reliability, and content and construct validity. Another study to evaluate the generalizability and relevance of the PROMIS-PF item bank involved the recruitment of 288 patients undergoing surgery for common foot and ankle problems [17]. Face validity was demonstrated through expert review by a panel of 6 foot and ankle surgeons. Construct validity was demonstrated through correlation analysis between PROMIS-PF and PROMIS-Pain scores and t-tests between groups of patients classified by disease severity [17]. Finally, a study by Voshaar and colleagues [18] assessed the content and construct validity of the PROMIS-PF item bank and 20-item short form in patients (*N* = 690) with rheumatoid arthritis. Content validity was established by linking the PROMIS-PF items to the International Classification of Functioning, Disability and Health (ICF) core set for rheumatoid arthritis. Construct validity was demonstrated by correlating PROMIS-PF scores with other clinical and patient-reported outcome measures (HAQ-Disability Index and SF-36).

The aim of this study was to summarize the evidence gained from qualitative patient interviews [11] to select the relevant items and demonstrate the content validity of a customized PROMIS-PF short form for patients with TGCT. This work supports the selection and relevance of the PF lower extremity scale as an endpoint for clinical trials of treatments for TGCT.

## Methods

The study included two primary components: (i) a qualitative study to gather input directly from patients on the impacts of their disease, and (ii) identification and selection of the key items to be used to measure physical functioning, sourced from the PROMIS-PF item database. Each of these components is described in greater detail below. As described in a previous publication, clinical experts (SB, JH, RL, WT) provided helpful input throughout the study [11].

### Patient interviews

This was a cross-sectional, qualitative interview study involving semi-structured interviews and completion of self-administered questionnaires [11]. Participants were recruited from private clinical sites, online blogs communicating their TGCT diagnosis, or via disease-related websites. Participants were eligible if they were able to participate in a one-on-one interview over the phone or in-person, male or female ≥18 years old, had histologically confirmed TGCT, able to read and speak English, and were willing and able to provide written informed consent prior to the interview. Participants were excluded if they had significant cognitive impairment, hearing difficulty, visual impairment, severe psychopathology, or any systemic or local illness or medical condition that could significantly interfere with the participant’s perception of TGCT specific symptoms.

#### Interview procedures

The semi-structured interview guide included two main parts. The first part involved concept elicitation to identify the key relevant symptom concepts and the impacts of these symptoms as experienced by patients. The initial open-ended questions asked participants to talk about the location of their tumor, the diagnostic process, treatments, symptoms they had experienced (description, frequency, variability, relationship with pain), and impacts they had noticed. The second part included a cognitive interview that allowed the patient to provide feedback on the content and their understanding of the PROMIS-PF items. This included questions about the relevance, instructions, item content, recall period, and response options.

As part of the cognitive debriefing, participants were provided with two checklists of items from the PROMIS-PF item bank, and they were asked to review each list and indicate which of the PROMIS-PF items were relevant to them in the context of their tumor-related impacts. The first checklist included items that were potentially relevant only to those with lower extremity tumors, and the second checklist included items potentially relevant to those with a tumor in any location. The purpose of completing these checklists was to quickly and easily identify key PROMIS-PF items that were relevant to a substantial number of the participants.

#### Analysis

Descriptive statistics (mean, standard deviation, frequency) for sociodemographic and clinical data were used to characterize the sample. Qualitative data collected in both the concept elicitation and cognitive interviewing portions of the one-on-one semi-structured patient interviews was reviewed, and any information related to physical functioning as described by the patients was extracted. Finally, the frequency and proportion of participants that endorsed items on the PROMIS-PF checklists were calculated. Based on the qualitative results and the PROMIS-PF checklist results, the most relevant PROMIS-PF items were selected for further evaluation. Considerations for narrowing the list of candidate items included: (i) relevance of the target concept for each item as evidenced by direct patient quotes, (ii) items that are frequently performed (e.g., daily) were preferred as the PROMIS-PF items do not include a recall period, and (iii) items that were specific were preferred (e.g., “stand unsupported for 10 minutes” would be selected over “stand for short periods of time).”

### Review of IRT-based PROMIS-PF item parameters

Based on the results of the qualitative interviews (i.e., patient input and checklist results), key PROMIS-PF items of interest were considered further. The statistical properties (i.e., the item-response theory based item slope and thresholds) of the PROMIS-PF candidate items were reviewed in order to identify item overlap or redundancy. The item-specific parameters are available on the PROMIS website and were estimated by the PROMIS developers using IRT. IRT models assume that a person’s level of physical function (e.g., high vs. low) will predict that person’s probability of endorsing each specific item. Once these item parameters are calibrated for each item in an item bank, they can be used to score any new response data from any subset of items.

The slope parameter (i.e., discrimination) refers to the ability of an item to differentiate between different levels of the latent trait. Generally, the higher the discrimination the better the item. Threshold describes the level of the latent trait at which the person is more likely to respond in the higher category than in the lower category. For example, threshold 2 is the level of the latent trait at which the person is likely to respond 2 (or higher) vs. 1. Each PROMIS item has five response options so there are four thresholds. Generally, while a wider spread of thresholds for an individual item is desirable, a range of thresholds across the whole instrument is best.

## Results

### Patient interviews

Face-to-face and telephone interviews were conducted with *N* = 20 participants (*n* = 15 females; *n* = 5 males). The mean age was 42.5, and ranged from 27 to 56 years. The locations of the participants’ tumors included the knee (*n* = 15; 75%), hip (*n* = 3; 15%), and ankle (*n* = 2; 10%). Patient-reported sociodemographic and clinical characteristics have been reported previously [11].

Participants described a range of symptoms in the concept elicitation portion of the interviews, many of them spontaneously. Pain and swelling were the most commonly reported symptoms, each mentioned by a large majority of the participants; 80% and 85%, respectively [11]. Stiffness, reduced range of motion, and instability or giving out/giving way were also commonly reported symptoms: 75%, 65%, and 65%, respectively [11]. Participants consistently reported that their symptom experiences impacted their physical functioning.

Quotes that were used to identify relevant PROMIS-PF lower extremity items are shown in Table 1. Participants were not exposed to the content of the PROMIS-PF items prior to the qualitative portions of the interview (i.e., the PROMIS-PF checklist was completed after the interview). There was high concordance between the PROMIS-PF items and the examples and descriptions provided by interview participants. The most commonly described challenge and impact for lower extremity tumor participants, described by all but two (18/20), dealt with the navigation of stairs (Item: *Are you able to go up and down stairs at a normal pace?*). In addition, nearly all lower extremity tumor participants (17/20) discussed their difficulties with being able to stand still for specific periods of time (Item: *Are you able to stand for 1 hour?*). Similarly, 17 of 20 lower extremity tumor participants spoke of issues related to bending, kneeling, or stooping (Item: *Does your health now limit you in bending, kneeling, or stooping?*). Over half of the participants (13/20) with lower extremity tumors commented about challenges regarding the length or duration of walking (Item: *Are you able to go for a walk of at least 15 min?*), exercising (13/20) (Item: *Are you able to exercise for an hour?*), and the completion of chores around the house (12/20) (Item: *Does your health now limit you in doing moderate work around the house like vacuuming, sweeping floors or carrying in groceries?*).

**Table 1:** Selected patient quotes targeting PROMIS-PF item concepts

Are you able to go for a walk of at least 15 min?	*101–004: I used to be a pretty athletic person and it got to the point where even just walking a block, by the end of that time it just got so sore and the stiffness kind of got in.*
	*101–014: I’d like to do more walks. That isn’t going to happen, long distance walks or even for extended period of time.*
	*101–017: If I walk, you know, if I do a lot of walking, say like I’m at the store with my kids or something and I get into the car just from walking and then sitting, it, it will swell up right then, right then and there.*
Are you able to dress yourself, including tying shoelaces and buttoning up your clothes?	*101–016: If I’m trying to put shoes on or put pants on, um, just bending that knee to get the pants on or the boots, just a little stiff with a little bit of pain.*
	*101–017: Yeah I’m not wearing socks…in the winter time honestly I would have like my boyfriend, take my socks off for me, please. Yeah, so I mean, you know pretty much would help, you’re a child help me put them on. Can you help me put my socks on? Could you help me take ‘em off.*
	*101–021: It’s easy to put on my sock on my right foot, always has been, but the left foot—I mean, I—I’d like reach up and bend my leg and put on my sock on my right foot, kind of with my leg crossed over my other knee, but as that hip was giving me trouble, I couldn’t lift up, and I still can’t because of the fake joint. I can’t put that leg over to—to reach and put the sock on, so I would have to try to prop it up on the bed and then reach over to get the sock on, so it was a—it was a more involved process to put the sock on the foot with the PVNS going on on that side of my body.*
Does your health now limit you in going OUTSIDE the home, for example to shop or visit a doctor’s office?	*101–011: I guess the worst part is, you know, if we do have to drive somewhere, um, I am not going to be able to go and do something right away if we, you know, once we get somewhere because it can take several hours until I’m really able to walk well if it gets bad.*
	*101–017: If I do any walking, you know, grocery store, by the time I finish with the grocery store I’m pretty much, burning up I would say. I’m not as active as I used to be at one time, I—I’m just, my activity level has really been limited since all this came about.*
	*101–019: I can’t do—I can’t do biking on a bicycle, I can’t go hiking, I can’t do anything that I used to do. I am just—going to the mall, I can’t even do that, it’s just—sorry. I tried to go to the mall twice and I—I just am in so much pain, I just have to leave.*
Does your health now limit you in doing heavy work around the house like scrubbing floors, or lifting or moving heavy furniture?	*101–001: I don’t do heavy household chores now because I can’t handle it pain wise because I have to have the energy for my daughter. So I conserve my energy and my pain. So I’m not doing that.*
	*101–011: I definitely have more problems, especially doing yard work kind of things. Um, the heavier type stuff.*
	*101–021: If I’m out mowing the yard, it’s—it’s a challenge as the old mower bag gets full of grass for me to push it. I definitely can’t push it as fast as I used to. I cannot finish the yard in 20–25 min. It takes me 45 now, and I think some of that has to do with the musculature around where that tumor was*
Are you able to push open a heavy door?	*101–004: Right before the surgery. I just wouldn’t do anything that required pushing or pulling.*
	*101–019: Any pushing—pulling is very painful. It just feels like, like I know using that part of my leg it just is—it just can be so painful.*
Are you able to carry a heavy object (over 10 pounds/5 kg)?	*101–001: This morning I was on the fourth floor of the building and it had a false fire alarm, so I had to walk down four flights of stairs and walk back up four flights of stairs. …..It was very, very, very painful and I was carrying my daughter, which she weighs 25 pounds, so –*
	*101–004: My daughter was young enough that she wanted to be held and carried and I couldn’t because it would shift my balance off so much that if I did have any sort of, you know, instability, I was terrified that I would drop her.*
Does your health now limit you in doing moderate work around the house like vacuuming, sweeping floors or carrying in groceries?	*101–002: My house is [number] square foot and I have to stop and I only have carpeting in – I have carpeting everywhere but two rooms, so I have to do it in sections. I can’t do it continuously. I have to stop and rest in between.*
	*101–012: I knew it was happening a lot. I--actually after it healed about two weeks later it--I—that really showed me how much I was compensating because the little things like bending down in the grocery store to get a product off the lower shelf, um, and cleaning around the house. I just didn’t realize I was compensating for the knee.*
	*101–014: Ah, like vacuuming. That doesn’t work too well because you plant yourself and you kind of twist to do that. At times like after surgeries and for quite some time, I will wear like my heavy duty boot which does not let me bend it. I ask other people to do it or I stand and plant myself and do more with like my arms instead of my legs. And I have compensated or get other people to do it.*
Does your health now limit you in lifting or carrying groceries?	*101–004: So, you know, even with grocery shopping, I really have to watch where my center of gravity is and, you know, again, pivoting on the bad leg, um, try to avoid doing that, especially carrying the extra weight of the groceries.*
Are you able to go up and down stairs at a normal pace?	*101–001: I have a pretty hard time going down stairs…. And, I mean, just going down just a couple stairs gives me difficulty because if my pain is enough, I have to take one like just one stair at a time, you know what I mean?.... I do have difficulty going up the stairs. Um, unless I have a rail to hang onto, and then maybe it would be a little lower.*
	*101–008: I can climb them and I can climb them one foot after the other, but I can feel the pain in the knee when I do that…. Stairs are probably one of the things where I notice it a lot.*
	*101–019: I’ve been trying to walk up and down the stairs like, you know, like normal. You know, like you go your left foot, then your right foot – that kind of thing, instead of just going one leg, one leg, one leg, up and down. And going down has the tendency to uh—I just have difficulty bending my leg. I just think it’s worse going down, trying to with my leg.*
Are you able to carry a laundry basket up a flight of stairs?	*101–004: Um, a lot of times you’re carrying or pushing a cart or carrying a basket, and anytime I have to kind of, not just me, but I have to like push a cart or carry a basket, it just adds a little bit more, um, so it is just – I don’t want to say it is exceedingly difficult, but I definitely am more focused to make sure that I don’t shift my weight wrong or do something like that.*
	*101–011: Carrying laundry up and, you know, carrying loads of laundry up and down the stairs that type of thing…. I definitely have issues, um, doing those types of things.*
Are you able to stand for one hour?	*101–001: My biggest issue is just standing still, you know, just putting all my weight…. It’s just – it happens all the time if I stand in one spot for more than like a couple minutes. I mean, probably within 30 s of standing – just standing, I start feeling like major discomfort.*
	*101–005: I mean, I could stand for 30 min at a time, but it was not comfortable and I – it would, um, be painful and be a lot more swollen and full of fluid and all of that.*
	*101–019: You know I can’t even stand for very long. When I go to church, I stand for a little bit and then I just have to sit down.*
Does your health now limit you in bending, kneeling, or stooping?	*101–010: If I drop something on the floor I will try to find a way to pick it up with my toes. You know, bending at the hips instead of the knee because if I—if I bend with the knee that hurts a lot.*
	*101–017: So I don’t have a lot or range of motion on my knee, so I can’t bend down on my knees or get on my knees and, you know, kneel down or anything like that. I’m not able to do that. I can’t put any pressure on my knees and bending it, you know, the range of motion on my knee is not very good at all.*
	*101–018: I would never think of bending to the floor using my hips so I think of bending to the floor as squatting down, bending my knees to the floor and that is extremely painful or probably the most painful.*
Are you able to exercise for an hour?	*101–001: I had to stop, um, running. I had to put it up as much as I could when I wasn’t working. So basically like as soon as I got home from work, I’d just sit on the couch and have it elevated, so I couldn’t do much. Definitely limited my activity.*
	*101–010: I used to work out on my light elliptical machine and, uh, exercise bikes and stuff. I can’t do that anymore just because—well for the exercise bike that much movement makes the knee hurt.*
	*101–011: I mean, I can’t run, I can’t, do any, you know, high impact kind of activities. I have a hard time even bicycling because of the range of motion, so, but I can ride a bike if I’m really careful, then I can ride. Um, but yeah, it affects my things I can do every single day.*

Results of the PROMIS-PF checklist exercise were complementary to the descriptions provided by patients (Additional file 1: Table S1). Of the 48 items on the list of items potentially relevant to individuals with a tumor of any location, 10 items were endorsed by the majority of participants (range: 50%—80%). The two most commonly endorsed items (80%) were *Participate in active sports?* and *Doing vigorous activities, such as run*.

Among the 39 items administered to the participants with lower extremity tumors, 20 items were endorsed by the majority of participants (50% - 90%). Some common items among the lower extremity participants included: *Are you able to go up and down stairs at a normal pace?* (85%); *Does your health now limit you in bending, kneeling, or stooping?* (80%); *Are you able to stand for 1 hour?* (80%).

### Review of IRT-based PROMIS-PF item parameters

The statistical properties of the individual PROMIS candidate items, which were selected based on direct patient input during the qualitative patient interviews and from the item checklist exercise, were reviewed in order to inform item overlap and/or redundancy. For concepts where multiple relevant items were available in the PROMIS-PF item bank, items were preferred if they were typically performed daily, and were less subject to variable interpretation. Candidate items with maximal slopes (range: 2.96–4.399) and appropriately targeted thresholds (range: − 3.29–0.31) were selected (Table 2). This yielded 13 for the lower extremity scale. As an example of the item-selection process, both “Are you able to run errands and shop?” and “Does your health now limit you in going OUTSIDE the home, for example to shop or visit a doctor’s office?” were items that were relevant to participants based on the qualitative results. However, they assess essentially the same concept, therefore only one was appropriate for inclusion. The latter was selected because it is more specific, was easier to translate, and the IRT parameters encompassed a wider range of thresholds.

**Table 2:** PROMIS-PF items and parameters

PROMIS Physical Functioning Item	Item Wording	IRT Parameters^a,b^				
		Slope	Threshold 1	Threshold 2	Threshold 3	Threshold 4
Lower Extremity						
PFA23	Are you able to go for a walk of at least 15 min?	4.28753	− 1.9146	− 1.5792	− 1.1944	− 0.6814
PFA21	Are you able to go up and down stairs at a normal pace?	4.23685	−1.885	−1.4805	− 1.0464	− 0.3953
PFA10	Are you able to stand for one hour?	3.37529	−1.7469	−1.3522	− 0.9182	− 0.3361
PFA3	Does your health now limit you in bending, kneeling, or stooping?	2.94958	−2.25	−1.2635	− 0.563	0.029
PFA42	Are you able to carry a laundry basket up a flight of stairs?	4.39902	−1.8357	−1.4509	− 1.0267	− 0.4249
PFB1	Does your health now limit you in doing moderate work around the house like vacuuming, sweeping floors or carrying in groceries?	4.38889	−2.4474	−1.6384	−1.076	−0.632
PFA5	Does your health now limit you in lifting or carrying groceries?	4.13549	−2.3684	−1.6186	−1.0365	−0.5334
PFA12	Are you able to push open a heavy door?	3.45638	−2.7039	−2.0429	−1.52	−0.7504
PFA14r1	Are you able to carry a heavy object (over 10 pounds/5 kg)?	3.43	−2.21	−1.74	−1.25	−0.66
PFB54	Does your health now limit you in going OUTSIDE the home, for example to shop or visit a doctor’s office?	3.39556	−3.286	−2.2698	−1.6877	−1.1944
PFA16r1	Are you able to dress yourself, including tying shoelaces and buttoning up your clothes?	3.37	−3.14	−2.56	−1.91	−1.24
PF4	Does your health now limit you in doing heavy work around the house like scrubbing floors, or lifting or moving heavy furniture?	4.39902	−1.5594	−0.9773	−0.4150	0.0586
PFA13	Are you able to exercise for an hour?	2.95971	−1.4114	−0.9872	−0.415	0.3052

^a^Slope aka “discrimination” refers to the ability of the item to differentiate between different levels of the latent trait. Generally, the higher the discrimination the better the item as it indicates that people with a higher latent trait are much more likely to respond in the higher category

^b^Threshold in the Graded Response Model describes the level of the latent trait at which the person with that latent trait is more likely to respond in the higher category than in the lower category. i.e. Threshold 1 – the level of the latent trait at which the person is likely to respond 1 (or higher) vs. 0; Threshold 2 – the level of the latent trait at which the person is likely to respond 2 (or higher) vs. 1; Threshold 3 – the level of the latent trait at which the person is likely to respond 3 (or higher) vs. 2 and Threshold 4 – the level of the latent trait at which the person is likely to respond 4 vs. 3. There are no responses higher than 4 possible for 5-category items with item scores ranging from 0 to 4. Generally, while a wider spread of thresholds for individual item is desirable, a mixture of threshold levels across the whole instrument is best

## Discussion

The PROMIS-PF items identified for inclusion in the lower extremity scale were selected based on the combined evidence across all areas of research used in this study. Input from clinical experts [11], direct patient interviews, the results of a PROMIS-PF checklist exercise, and information on the individual PROMIS-PF item properties were used to identify the most relevant, broadly applicable and appropriate items with which to measure impacts on physical functioning in patients with lower extremity TGCT tumors. The use of the PROMIS-PF IRT parameters in particular, is a novel application of instrument methodology which enhances confidence in the validity of instruments in rare diseases such as TGCT where it might be difficult to recruit sufficient patients for traditional validation.

This study did seek to gain input from individuals with upper extremity tumors. However, as the majority of TGCT tumors occur in the lower extremities, recruitment of individuals with upper extremity tumors was challenging, and only two participants were enrolled. Though input from participants with upper extremity tumors was limited, and the results of the PROMIS-PF checklist exercise was only marginally informative among these patients, it is important to note that the review of the qualitative interview transcripts from these upper extremity patients provided highly specific and relevant information that was consistent across the two. For example, there were five PROMIS-PF concepts that were discussed as PF impacts by both upper extremity participants (i.e., *exercise for an hour, moderate work around the house, lifting or carrying groceries, carry a heavy object,* and *push open a heavy door*). These limited data represent a valuable contribution to our knowledge on this important subgroup from a rare disease population, however, additional studies of patients with upper extremity tumors is an area for future research.

There are multiple strengths in the use of the PROMIS-PF scales in the TGCT patient population. First, TGCT tumors can be found in either the upper extremities or lower extremities, and the PROMIS-PF item bank provides the opportunity to include measurement of the impacts of tumors regardless of location in a way that is not possible with other measures. Second, the IRT scoring approach of PROMIS allows for item reduction and customization of scales that are unidimensional and not excessively redundant. In addition, because the PROMIS-PF items were calibrated together, the validity of the item bank has been established, all items are considered to be on the same metric, and item parameters do not have to be recalibrated in each patient population [19, 20]. The physical functioning scores for each participant, regardless of tumor location, can be scored on the same physical functioning metric and analyzed together.

The content validity work reported herein is consistent with an important goal of the PROMIS initiative, which is application of the PROMIS item banks across patient populations [12]. A perspective paper by Magasi and colleagues [21], emphasized the importance of content validity in the PROMIS items across patient populations. In this paper, the working group advocated for meticulously documented qualitative and quantitative methods for the evaluation of content validity. Further, the group recommended empirical evaluation of generalizability of content validity across applications, and use of generic measures (i.e., PROMIS item banks) as the foundation for PRO assessment [21].

In addition to the work done in lower extremity orthopedic [16, 17] and arthritis patient populations [18], Garcia and colleagues [22] have highlighted actions of the National Cancer Institute (NCI) to assure content validity and application of the PROMIS item banks to cancer patients and survivors. NCI supported the data collection for item calibration and norming from 2000 patients with cancers of various types. Data collection included administration of the PROMIS item banks to 500 patients recruited from cancer clinics and tumor registries, and 1500 patients across the continuum of cancer care [22]. In addition, NCI placed an emphasis on the achievement of content and construct validity through the inclusion of domain expert and patient input via focus groups or cognitive interviews to enhance the cancer relevance of the five PROMIS domains [23].

There are limitations to the work herein that deserve mention. Recruitment of patients with a rare disease can be extremely challenging. This study was unable to recruit a sample of patients representing all bodily locations that can be affected by TGCT. For example, no participants experienced a tumor in the jaw or spine, and only two participants had tumors located in the upper extremities. As a consequence, there are some assumptions made about the nature and extent of the impacts of TGCT on physical functioning for patients with tumors in those locations. In addition, our analysis of the qualitative interview data relevant to the specific PROMIS-PF items was not an a priori goal of this work. Had we specifically probed during the interviews on all concepts in the PROMIS-PF it is possible that participant feedback would have been supportive of fewer or more items, yet those results would have arguably been vulnerable to investigator/interviewer and responder bias [24]. It is important to note that this study demonstrates the content validity of the lower extremity items that were included in the short forms; it does not address whether any additional items should have been included. In other words, the data demonstrate the relevance of the included items, but whether other additional items of relevance should have been included is not directly addressed by the study design. Finally, the PROMIS items are not presented with a specific recall period (e.g., 7-day recall) [15]. This was addressed in candidate item selection for this study by focusing on generalizable, common, daily activities and tasks, but may be viewed by some as a limitation of the PROMIS-PF item bank.

The PROMIS-PF items identified through this study were included as outcome measures in a Phase III clinical trial of pexidartinib (a small molecule kinase inhibitor of CSF1R) in patients with TGCT (NCT02371369). The process to identify these subscales was consistent with guidance on PRO measures issued by the FDA and European Medicines Agency (EMA) [25, 26]. The methods reported in this study are recommended for those aiming to identify relevant PROMIS-PF items for other disease indications where patients may be difficult to recruit and/or the patient population may have heterogeneous manifestations in the concept being measured. The use of the PROMIS item banks to develop and score custom forms based on the IRT-item parameters estimated during the development and calibration of the PROMIS item banks assumes that there are no condition-by-item interactions (i.e., measurement invariance). Prior research across several other disease areas has shown instances of differential item functioning to be fairly rare, and to have negligible effects on the calculated scores [27, 28].

## Conclusions

Considerable background and qualitative research among patients with TGCT was undertaken to identify impacts on physical functioning. The results of clinician interviews, a qualitative interview study, and review of PROMIS-PF item parameters were used to identify relevant items for the measurement of physical functioning among patients with this rare disease. Two PROMIS-PF scales were created, tailored to patients with either upper or lower extremity tumors respectively, with overlap in items across the two scales. These scales were incorporated into a phase III clinical trial (ENLIVEN; NCT02371369). There is substantial evidence from prior research to support the content validity of the PROMIS-PF items generally. The qualitative study conducted among patients with TGCT [11] further supports the content validity of the PROMIS-PF items in this patient population specifically.

## Additional file

Additional file 1: **Table S1.** PROMIS-PF Checklist Results: Participant Endorsed Relevant Items. (DOCX 20 kb)

## Abbreviations

BPI: Brief Pain Inventory
CAT: Computer adaptive test
CSF1: Colony-stimulating factor 1
CSF1R: Colony-stimulating factor 1 receptor
EMA: European Medicines Agency
FDA: Food and Drug Administration
GCT-TS: Giant cell tumors of the tendon sheath
HAQ: Health Assessment Questionnaire
HRQL: Health-related quality of life
ICF: International Classification of Functioning, Disability and Health
IRT: Item response theory
NCI: National Cancer Institute
NRS: Numerical rating scale
PF: Physical function
PRO: Patient-reported outcome
PROMIS: Patient-Reported Outcomes Measurement Information System
PVNS: Pigmented villonodular synovitis
QOL: Quality of life
SF-36: 36-Item Short Form Health Survey
TGCT: Tenosynovial giant cell tumor
UE: upper extremity
WOMAC: Western Ontario and McMaster Universities Osteoarthritis Index
