# Supplementary material for: The measurement of physical functioning among patients with Tenosynovial Giant Cell Tumor (TGCT) using the Patient-Reported Outcomes Measurement Information System (PROMIS)
Source: J Patient Rep Outcomes. 2019 Feb 4;3:6. doi: 10.1186/s41687-019-0099-0 (PMC6360193; doi:10.1186/s41687-019-0099-0)
Supplement: Supplementary file 1 — Table S1. PROMIS-PF Checklist Results: Participant Endorsed Relevant Items. (DOCX 20 kb) [file 41687_2019_99_MOESM1_ESM.docx]

Additional file 1: Table S1. PROMIS-PF Checklist Results: Participant Endorsed Relevant Items

| Participant ID Tumor Location | All | Upper | Lower |
| --- | --- | --- | --- |
| Participate in active sports? | 17 (81.0%) | 1 (50.0%) | 16 (84.2%) |
| Doing vigorous activities, such as run. | 17 (81.0%) | 1 (50.0%) | 16 (84.2%) |
| Doing 8 hours of physical labor? | 16 (76.2%) | 0 (0.0%) | 16 (84.2%) |
| Doing strenuous activities...? | 16 (76.2%) | 1 (50.0%) | 15 (78.9%) |
| Doing 2 hours of physical labor? | 15 (71.4%) | 1 (50.0%) | 14 (73.7%) |
| Exercise for an hour? | 14 (66.7%) | 1 (50.0%) | 13 (68.4%) |
| Doing heavy work around the house...? | 13 (61.9%) | 0 (0.0%) | 13 (68.4%) |
| Your physical activities (walking,...?) | 13 (61.9%) | 0 (0.0%) | 13 (68.4%) |
| Doing moderate activities | 12 (57.1%) | 1 (50.0%) | 11 (57.9%) |
| Carry a laundry basket up a flight...? | 11 (52.4%) | 1 (50.0%) | 10 (52.6%) |
| Chores | 10 (47.6%) | 0 (0.0%) | 10 (52.6%) |
| Do yard work, like raking leaves. | 10 (47.6%) | 0 (0.0%) | 10 (52.6%) |
| Push open a heavy door? | 9 (42.9%) | 2 (100.0%) | 7 (36.8%) |
| Doing moderate work around the house,... | 9 (42.9%) | 0 (0.0%) | 9 (47.4%) |
| Carry a heavy object (over 10 pounds)? | 9 (42.9%) | 1 (50.0%) | 8 (42.1%) |
| Exercise for half an hour? | 9 (42.9%) | 1 (50.0%) | 8 (42.1%) |
| Run errands and shop? | 9 (42.9%) | 1 (50.0%) | 8 (42.1%) |
| Carry 2 bags of groceries 100 yards? | 8 (38.1%) | 1 (50.0%) | 7 (36.8%) |
| Get in and out of a car? | 8 (38.1%) | 0 (0.0%) | 8 (42.1%) |
| Bend down and pick up clothing? | 8 (38.1%) | 0 (0.0%) | 8 (42.1%) |
| Your daily physical activities...? | 7 (33.3%) | 0 (0.0%) | 7 (36.8%) |
| Move a chair from one room to another? | 6 (28.6%) | 0 (0.0%) | 6 (31.6%) |
| Lifting or carrying groceries? | 6 (28.6%) | 1 (50.0%) | 5 (26.3%) |
| Put on and take off socks? | 5 (23.8%) | 0 (0.0%) | 5 (26.3%) |
| Reach and get down a 5-pound object...? | 5 (23.8%) | 1 (50.0%) | 4 (21.1%) |
| Turn in bed | 5 (23.8%) | 0 (0.0%) | 5 (26.3%) |
| Wash dishes, pots, and utensils..? | 4 (19.0%) | 0 (0.0%) | 4 (21.1%) |
| Carry a shopping bag or briefcase? | 4 (19.0%) | 1 (50.0%) | 3 (15.8%) |
| Tie your shoelaces? | 4 (19.0%) | 0 (0.0%) | 4 (21.1%) |
| Getting in and out of the bathtub | 4 (19.0%) | 0 (0.0%) | 4 (21.1%) |
| Dress yourself, including tying shoelace | 3 (14.3%) | 0 (0.0%) | 3 (15.8%) |
| Going Outside the home, to shop | 3 (14.3%) | 0 (0.0%) | 3 (15.8%) |
| To take a tub bath? | 3 (14.3%) | 0 (0.0%) | 3 (15.8%) |
| Get on and off toilet? | 2 (9.5%) | 0 (0.0%) | 2 (10.5%) |
| Be out of bed most of the day? | 2 (9.5%) | 0 (0.0%) | 2 (10.5%) |
| Bathing or dressing yourself? | 2 (9.5%) | 0 (0.0%) | 2 (10.5%) |
| Make abed, including spreading or tucking | 2 (9.5%) | 0 (0.0%) | 2 (10.5%) |
| Change a light bulb overhead? | 1 (4.8%) | 0 (0.0%) | 1 (5.3%) |
| Putting a trash bag outside | 1 (4.8%) | 0 (0.0%) | 1 (5.3%) |
| Pull on trousers? | 1 (4.8%) | 0 (0.0%) | 1 (5.3%) |
| To transfer from a bed to a chair & back | 1 (4.8%) | 0 (0.0%) | 1 (5.3%) |
| Push open a door after turn the knob? | 1 (4.8%) | 1 (50.0%) | 0 (0.0%) |
| Wash and dry your body? | 1 (4.8%) | 0 (0.0%) | 1 (5.3%) |
| Get in and out bed? | 1 (4.8%) | 0 (0.0%) | 1 (5.3%) |
| Taking care of personal needs | 0 (0.0%) | 0 (0.0%) | 0 (0.0%) |
| Water a house plant | 0 (0.0%) | 0 (0.0%) | 0 (0.0%) |
| Taking a shower? | 0 (0.0%) | 0 (0.0%) | 0 (0.0%) |
| Wipe yourself after using the toilet? | 0 (0.0%) | 0 (0.0%) | 0 (0.0%) |
|  |  |  |  |
| Run 10 miles? | 18 (90.0%) | 0 (0.0%) | 18 (90.0%) |
| Run 5 miles? | 18 (90.0%) | 0 (0.0%) | 18 (90.0%) |
| Run for 2 miles? | 18 (90.0%) | 0 (0.0%) | 18 (90.0%) |
| Run fast past for 2 miles? | 18 (90.0%) | 0 (0.0%) | 18 (90.0%) |
| Go up and down stairs | 17 (85.0%) | 0 (0.0%) | 17 (85.0%) |
| Run 100 yards | 17 (85.0%) | 0 (0.0%) | 17 (85.0%) |
| Run a short distance? | 16 (80.0%) | 0 (0.0%) | 16 (80.0%) |
| Kneel on the floor? | 16 (80.0%) | 0 (0.0%) | 16 (80.0%) |
| Bend, kneel or stoop? | 16 (80.0%) | 0 (0.0%) | 16 (80.0%) |
| Stand for one hour | 15 (75.0%) | 0 (0.0%) | 15 (75.0%) |
| Squat | 15 (75.0%) | 0 (0.0%) | 15 (75.0%) |
| Jump and down | 15 (75.0%) | 0 (0.0%) | 15 (75.0%) |
| Hiking a couple of miles? | 15 (75.0%) | 0 (0.0%) | 15 (75.0%) |
| Stand from an armless straight chair | 14 (70.0%) | 0 (0.0%) | 14 (70.0%) |
| Climbing several flights of stairs? | 13 (65.0%) | 0 (0.0%) | 13 (65.0%) |
| Climb up 5 flights of stairs? | 13 (65.0%) | 0 (0.0%) | 13 (65.0%) |
| Get up off the floor from lying on your | 11 (55.0%) | 0 (0.0%) | 11 (55.0%) |
| Stand from a low soft couch? | 11 (55.0%) | 0 (0.0%) | 11 (55.0%) |
| Walk more than 1 mile? | 11 (55.0%) | 0 (0.0%) | 11 (55.0%) |
| Stand straight | 10 (50.0%) | 0 (0.0%) | 10 (50.0%) |
| Go for a walk of at least 15 minutes | 9 (45.0%) | 0 (0.0%) | 9 (45.0%) |
| Step up and down curbs | 9 (45.0%) | 0 (0.0%) | 9 (45.0%) |
| Walk at normal speed? | 9 (45.0%) | 0 (0.0%) | 9 (45.0%) |
| Climbing one flight of stairs? | 9 (45.0%) | 0 (0.0%) | 9 (45.0%) |
| Climb up five steps? | 8 (40.0%) | 0 (0.0%) | 8 (40.0%) |
| Stand unsupported for 30 minutes | 8 (40.0%) | 0 (0.0%) | 8 (40.0%) |
| Reach into low cupboard? | 8 (40.0%) | 0 (0.0%) | 8 (40.0%) |
| Walk several hundred yards? | 8 (40.0%) | 0 (0.0%) | 8 (40.0%) |
| Stand up on tiptoes? | 7 (35.0%) | 0 (0.0%) | 7 (35.0%) |
| Walk a block on flat ground | 6 (30.0%) | 0 (0.0%) | 6 (30.0%) |
| Walk up and down 2 steps? | 6 (30.0%) | 0 (0.0%) | 6 (30.0%) |
| Going for a short walk? | 5 (25.0%) | 0 (0.0%) | 5 (25.0%) |
| Walk 100 yards? | 5 (25.0%) | 0 (0.0%) | 5 (25.0%) |
| Stand without losing balance for mins | 4 (20.0%) | 0 (0.0%) | 4 (20.0%) |
| Walk about the house? | 4 (20.0%) | 0 (0.0%) | 4 (20.0%) |
| Stand for short periods of time | 3 (15.0%) | 0 (0.0%) | 3 (15.0%) |
| Stand unsupported for 10 minutes | 2 (10.0%) | 0 (0.0%) | 2 (10.0%) |
| Get out of bed and into chair | 1 (5.0%) | 0 (0.0%) | 1 (5.0%) |
| Sit on the edge of the bed | 0 (0.0%) | 0 (0.0%) | 0 (0.0%) |
|  |  |  |  |
| Lift 10lbs above your shoulders? | 1 (50.0%) | 1 (50.0%) | 0 (0.0%) |
| Reach into a high cupboard | 1 (50.0%) | 1 (50.0%) | 0 (0.0%) |
| Use hammer to pound a nail | 1 (50.0%) | 1 (50.0%) | 0 (0.0%) |
| Pull heavy objects (10lbs) towards self | 1 (50.0%) | 1 (50.0%) | 0 (0.0%) |
| Hold a plate of food | 1 (50.0%) | 1 (50.0%) | 0 (0.0%) |
| Press with your index finger? | 1 (50.0%) | 1 (50.0%) | 0 (0.0%) |
| Cut food using eating utensils | 0 (0.0%) | 0 (0.0%) | 0 (0.0%) |
| Open previously opened jars | 0 (0.0%) | 0 (0.0%) | 0 (0.0%) |
| Open a can with a hand can opener | 0 (0.0%) | 0 (0.0%) | 0 (0.0%) |
| Wash your back | 0 (0.0%) | 0 (0.0%) | 0 (0.0%) |
| Open and close a zipper | 0 (0.0%) | 0 (0.0%) | 0 (0.0%) |
| Dry your back with a towel | 0 (0.0%) | 0 (0.0%) | 0 (0.0%) |
| Turn a key in a lock | 0 (0.0%) | 0 (0.0%) | 0 (0.0%) |
| Write with a pen or pencil | 0 (0.0%) | 0 (0.0%) | 0 (0.0%) |
| Put on a shirt or blouse | 0 (0.0%) | 0 (0.0%) | 0 (0.0%) |
| Peel fruit | 0 (0.0%) | 0 (0.0%) | 0 (0.0%) |
| Bend or twist back | 0 (0.0%) | 0 (0.0%) | 0 (0.0%) |
| Brush teeth | 0 (0.0%) | 0 (0.0%) | 0 (0.0%) |
| Button your shirt | 0 (0.0%) | 0 (0.0%) | 0 (0.0%) |
| Change the bulb in a table lamp | 0 (0.0%) | 0 (0.0%) | 0 (0.0%) |
| Shave your face or apply makeup? | 0 (0.0%) | 0 (0.0%) | 0 (0.0%) |
| Cut a piece of paper with scissors | 0 (0.0%) | 0 (0.0%) | 0 (0.0%) |
| Pick up coins from a table top | 0 (0.0%) | 0 (0.0%) | 0 (0.0%) |
| Pour liquid from a bottle into a glass? | 0 (0.0%) | 0 (0.0%) | 0 (0.0%) |
| Shampoo your hair | 0 (0.0%) | 0 (0.0%) | 0 (0.0%) |
| Tie a knot or bow? | 0 (0.0%) | 0 (0.0%) | 0 (0.0%) |
| Lift a full cup or glass to your mouth? | 0 (0.0%) | 0 (0.0%) | 0 (0.0%) |
| Open a new milk carton? | 0 (0.0%) | 0 (0.0%) | 0 (0.0%) |
| To open car doors? | 0 (0.0%) | 0 (0.0%) | 0 (0.0%) |
| Remove something from your back pocket? | 0 (0.0%) | 0 (0.0%) | 0 (0.0%) |
| Put on a pullover sweater? | 0 (0.0%) | 0 (0.0%) | 0 (0.0%) |
| Turn faucets on and off? | 0 (0.0%) | 0 (0.0%) | 0 (0.0%) |
| Trim your fingernails | 0 (0.0%) | 0 (0.0%) | 0 (0.0%) |
| Lift one pound to shoulder level? | 0 (0.0%) | 0 (0.0%) | 0 (0.0%) |
| Use your hands such as turning faucets | 0 (0.0%) | 0 (0.0%) | 0 (0.0%) |
| Squeeze a new tube of toothpaste | 0 (0.0%) | 0 (0.0%) | 0 (0.0%) |
